# Media composition modulates human embryonic stem cell morphology and may influence preferential lineage differentiation potential

**DOI:** 10.1371/journal.pone.0213678

**Published:** 2019-03-19

**Authors:** Linda Harkness, Xiaoli Chen, Marianne Gillard, Peter Paul Gray, Anthony Mitchell Davies

**Affiliations:** 1 Australian Institute for Bioengineering and Nanotechnology (AIBN), The University of Queensland, Brisbane, Australia; 2 Translational Cell Imaging Queensland, Institute of Health and Biomedical Innovation, Queensland University of Technology, Brisbane, Australia; University of Illinois at Chicago, UNITED STATES

## Abstract

Undifferentiated human embryonic stem cells have a distinct morphology (hESC). Changes in cell morphology during culture can be indicative of differentiation. hESC, maintained in diverse medias, demonstrated alterations in morphological parameters and subsequent alterations in underlying transcript expression and lineage differentiation. Analysis of morphological parameters showed distinct and significant differences between the undefined, less defined and Xeno-free medias while still maintaining pluripotency markers. This suggested that the less defined media may be creating dynamic instability in the cytoskeleton, with the cytoskeleton becoming more stabilised in the Xeno-free media as demonstrated by smaller and rounder cells. Examination of early lineage markers during undirected differentiation using d5 embryoid bodies demonstrated increased mesodermal lineage preference as compared to endodermal or ectoderm in cells originally cultured in Xeno-free media. Undefined media showed preference for mesoderm and ectoderm lineages, while less defined media (BSA present) demonstrated no preference. These data reveal that culture media may produce fundamental changes in cell morphology which are reflected in early lineage differentiation choice.

## Introduction

Human embryonic stem cells (hESC) are commonly defined by their ability to self renew and maintain their undifferentiated state. Investigations into individual hESC lines have demonstrated that substantial variability occurs between cell lines in their differentiation efficiency [1, 2]. As human pluripotent stem cells (hPSC) progress towards use in clinical applications and drug development [3–5] it becomes imperative to understand how exogenous factors, such as media composition, may influence cellular differentiation through affecting changes in morphological parameters. Reports have demonstrated that altering the physical microenvironment of PSC resulted in different cytoskeletal organisation and thus behaviour of self-renewal and lineage specification [6, 7].

A number of publications have reported genetic profiling and differentiation potential differences between individual hESC lines [2, 8–10]. Osafune et al [2] reported lineage specificity when comparing embryoid bodies (EBs) generated from 17 hESC lines; some cell lines demonstrated differentiation towards all three germ lineages, some two lineages (such as ectoderm and endoderm) and others one lineage. Comparison of these lines at early (22 or 26) and late (50 and 56) passage found that variability in culture technique and cellular senescence showed no differences in lineage choice. Tsankov et al [9] extended the work by Osafune et al and developed a ScoreCard assay with a revised gene dataset to determine core genes regulated during early differentiation (now available for Thermo-Fisher TaqMan hPSC ScoreCard). While lineage specificity was observed in these reports this appears to correlate with a genetic predisposition of the individual cell lines rather than changes imposed through modification in media formulation. Mikkola et al [8] examined hESC lines where differences were observed in the d10 EBs differentiation propensity with one of the lines failing to demonstrate endodermal differentiation. However, the genes examined were all later markers of differentiation and early differentiation was not investigated. Sun et al [11] suggested that hESC lines may maintain their pluripotency using individual mechanisms with variations in this balance impacting the cell lines lineage specification. Alterations in transcript profiles of key genes involved in the maintenance of pluripotency between individual cell lines have demonstrated significant enrichment in developmental processes [11, 12].

The first derivations of hESC were performed using mitotically inactivated mouse embryonic fibroblasts (MEFs) with DMEM or Knockout DMEM media for culture [13, 14]. This was followed with the use of animal matrices, such as Matrigel (MG), and culture in KnockOut DMEM media conditioned on MEFs (CM). While CM [15] was vital in developing hESC technologies it was undefined, had xenobiotic components and demonstrated batch variability thus eliminating its use for development of technologies for pharmacological applications or clinical practice. To exclude animal sourced components and improve safety for therapeutic applications, derivations were performed on human cells such as dermal and [16] foreskin [17] fibroblasts in CM, with culture continuing on human feeders or on MG in media conditioned on human cells or defined medias such as mTeSR1. The first defined mediums, mTeSR1 [18] and StemPro (Thermo Fisher Scientific), still retained protein components from bovine serum albumin (BSA) and were used in combination with either MG or recombinant molecules such as vitronectin or laminin as the matrix. These were followed by Xeno-free, chemically defined, medias such as Essential 8 [19] and StemMacs iPS-Brew XF (Miltenyi Biotec). When reporting these medias the focus, however, has been on demonstrating maintenance of pluripotency [20, 21] and morphological changes found between media have not been investigated.

The cytoskeleton is known to functionally respond to the cellular microenvironment with morphology and adherence of stem cells demonstrating influence on cell fate choices [22]. Nanotopographical surfaces have revealed directional differentiation of mesenchymal stromal (stem) cells (MSC) towards the osteogenic lineages by altering cell adhesion patterns [23]. hESC have also exhibited different adhesion patterns, such as changes in spatial distribution of adhesion-related molecules between pluripotent cells and their differentiated progeny [24]. hESC have also demonstrated a less developed cytoskeleton as compared to fibroblasts [25]. Liu et al suggested that changes in cellular adhesion and morphology of hPSC were linked to disruption of focal adhesion formation [26]. Chowdhury et al demonstrated that ESC are sensitive to stress generated by changes in cell spread [27]. Thus, understanding influences on adhesion and morphology can aid in understanding and controlling early differentiation processes [28, 29].

Digital light microscopy and analysis are routinely used to detect changes in cellular morphology and investigate protein expression in individual cells [30]. Changes in cellular morphology are important indicators of differentiation [31] and images can be analysed utilising proprietary or generic (such as CellProfiler, Broad Institute) software. These technologies present advanced multi parametric data analysis capabilities offering detailed morphometric information such as: nuclear and cell area, cell spread, cell roundness, nuclear displacement, nuclear intensity and intensity over the whole cell area. We used this technology in conjunction with qPCR to investigate underlying molecular events and the consequential changes in morphology when hESC were cultured in different media formulations.

During our studies, we observed distinct and measurable differences in hESC morphology when cell lines were maintained in different medias. We examined the effect of five different media on three hESC lines to establish how the cellular morphology and cytoskeletal organisation were influenced and if these changes could be related to lineage specification during early differentiation. All cell lines were established and cultured on MG in mTeSR1 prior to culture in 5 medias for 4–5 passages. Analysis of imaging demonstrated significant changes in nuclear and cell morphology between the different media and cytoskeletal transcript expression from a human focal adhesion microarray revealed significant changes between less defined and Xeno-free medias. Transcript expression of early lineage differentiation markers at day five EB formation suggested lineage specificity related to media. This may indicate that hESC cultures can be manipulated by a simple change of medium to predispose cells to specific lineage fate choice.

## Methods and materials

### Cell culture

Three hESC lines (MEL1, WA09, ESI-hES3), routinely cultured on MG (Corning) in mTeSR1 media (STEMCELL Technologies), were subcultured in 5 medias: mouse embryonic fibroblast conditioned media [15] (CM; in house), StemPro (SP; Life Technologies), Essential 8 (E8, Stem cell Technologies), StemMacs iPS-Brew XF (SM, Miltenyi Biotec) and mTeSR1 (mT), for, minimally, 4 passages. MG was used, at a 1:300 dilution in DMEM/F12, as the matrix for all cultures. Cell lines were routinely cultured on Falcon 6 well plates and passaged at sub-confluence using gentle cell dissociation reagent (STEMCELL Technologies) before reseeding in new media. Three independent experiments were analysed from each media condition.

To generate EBs, sub-confluent wells of MEL1 were harvested as small clumps using gentle cell dissociation reagent then cultured in 6 well low cluster plates (Corning) at a ratio of 3 wells of 2D culture to 1 well of EBs. Three independent experiments of EBs were cultured for 5 days in DMEM/F12 (Life Technologies) supplemented with 20% FBS (Life Technologies). EBs were washed in phosphate buffered saline without Ca2+ and Mg2+ (PBS2-) and stored as pellets at -80oC. 2D control samples were used as undifferentiated controls.

To generate a differentiated cell line, stromal-like cells, outgrowing from the edges of colonies in mT media, were isolated by selection (removal of matrix and hESC medium) and cultured in DMEM/F12 supplemented with 20% FBS. Cells were then cultured in SP, mT and E8 to establish if media composition also changed the morphology of differentiated cells. Stromal-like (ST) cells were passaged using TrypLE (Thermo Fisher Scientific) and plated in 6 well format for western blotting (DMEM/F12 only) or 96 wells for imaging (DMEM/F12, SP, mT and E8).

### Immunostaining

hESC were plated on precoated MG wells in 96-well Nunc F plates, ST cells were plated in the same format without the use of a precoating matrix. Following culture in the appropriate mediums, cells were washed in PBS2-, fixed in 10% formalin for 10–15 mins, before 3 x 5 minute washes in PBS2-. Wells were blocked using 4% FBS, 0.3% Triton-X100 in PBS2-, for 1 hour at room temperature (RT). Mouse anti-alpha tubulin (TUBA4A; clone DM1A; Sigma-Aldrich) was incubated overnight at 4oC in staining buffer (SB; PBS2-, 1% BSA, 0.3% Triton-X100). Mouse secondary Alexa-Fluor antibody (2 μg/ml) was diluted in SB and incubated for 2 hours at RT. Three washes of PBS2- were routinely performed between antibody incubation, secondary and counterstaining to remove excess stain. Wells were counterstained with 10 μg/ml Hoechst 33342 and 5 μg/ml Phalloidin-FITC, (Tetramethylrhodamine B isothiocyanate; Sigma-Aldrich) in PBS2- for 30 minutes at RT. Stained wells were maintained in PBS2- for imaging.

### Imaging and analysis

Cell morphology was assessed by High Content Imaging using the InCell Analyzer 2200 and IN Cell Investigator image analysis software (GE Healthcare). Morphological parameters were assessed in the nuclear and cytoplasmic compartments which were labelled with the fluorescent nuclear and cytoskeletal stains, Hoechst and Phalloidin respectively. Measured parameters included: nuclear area, nuclear intensity, nuclear displacement, cell area, nuclear to cell ratio, cell spread, cell roundness and cell intensity/area.

### Gene expression

Samples of hESC for RT-PCR were taken from each culture media condition (n = 3 independent experiments). RNA was isolated according to manufacturer’s instructions using an RNeasy purification mini kit (Qiagen) and included treatment using an RNase-free DNase set (Qiagen). Following RNA quantification on a Nanodrop, cDNA was generated, ccording to manufacturer’s instructions, using the RevertAid First Strand cDNA synthesis kit (Thermo Fisher Scientific). Ten μg/ml of cDNA were used for RT-PCR using 2x Fast SYBR green master mix (Applied Biosystems) on a 7500 fast real-time PCR machine (Applied Biosystems). Primer sets were purchased from either GeneWorks, (Thebarton, South Australia) or Integrated DNA Technologies (Singapore) and included POU5F1, SOX2, NANOG, DNMT3B, FERMT1, FERMT2 and FERMT2. Cycle threshold (CT) values were normalised to a geometric mean of four housekeeping genes (-Actin, B2M, HPRT1, TBP) to calculate the ΔΔCT and relative expression values.

Three biological replicates of MEL1, cultured in SP and E8 media, were processed on an RT2 Profiler PCR arrays for Human Focal Adhesions (Qiagen) using RT2 SYBR Green ROX qPRC mastermix (Qiagen). As above, a geometric mean was calculated using five housekeeping genes (ACTB, B2M, GAPDH, HPRT1, RPLP0) the ΔΔCT values calculated and the data, across the three biological replicates, analysed. Statistical significance for changes in transcript expression was set at over 1.5 fold difference in regulation with p < 0.05. A selection of primer sets for genes regulated over 1.5 fold were designed using NCBI Primer-Blast (CAV1, CAV2, ITGA9, ITGB5, ITGA2, AKT3, PAK3, ITGA6) and purchased from Integrated DNA Technologies. Three samples from each cell line, for each of the five mediums were tested for gene transcript levels for each primer set.

To analyse if MEL1 EBs derived from cultures in all five hESC medias demonstrated changes in lineage preference during differentiation, a custom RT2 PCR array was designed based on an early differentiation gene set curated from Tsankov et al [9]. Plates included positive and negative controls, four housekeeping genes (ACTB, B2M, HPRT1, RPLP0), two pluripotency markers (POU5F1 and SOX2), and seven genes for each lineage. Endoderm: FOXP2, FOXA1, CDH20, ELAVL3, PHOXB2, POU3F3, CABP7; mesoderm: ODAM, ESM1, HOPX, PLVAP, FOXF1, HAND1, HAND2; ectoderm: POU4F1, TRPM8, OLFM3, DMBX1, CDH9, NOS2, MYO3B. ΔΔCT and relative expression values were calculated from the CT and geometric means of the four housekeeping genes.

### Western blotting

Samples from each hESC line in each media and MEL1 ST cells (as positive control) were lysed in RIPA buffer supplemented with Complete 0 Mini EDTA free. Total protein concentration was measured using a nanodrop. Lysed proteins were mixed with 4x loading dye (Bio-Rad) and a reducing agent (Life Technologies) and heated to 70oC for 10 mins before loading onto 4–15% Mini-PROTEAN TGX stain-free Protein gels (Bio-Rad) in TRIS glycine running buffer (Bio-Rad). Gels were run for 30 minutes at 200V.

Trans-Blot Turbo system (Bio-Rad) was used for the transfer of proteins from the mini PROTEAN gel to the PVDF membrane with the transfer completed at 10 minutes. Membranes were blocked in PBS with 0.1% Tween (blocking buffer) and 5% milk powder for 1 hour with shaking. Primary antibodies (from a focal adhesion protein antibody sampler kit, Cell Signaling Technology) were diluted according to manufacturer’s instructions with blocking buffer and incubated with the membrane overnight at 4oC with rocking. The antibodies included: a-Actinin, FAK, Paxillin, Talin-1, Tensin 2, and Vinculin; alpha actin was utilised as a control. The membranes were washed 5 times with blocking buffer before goat anti-rabbit HRP secondary antibody (1:1000) was added in blocking buffer for 1 hr at RT with rocking. Antibody detection was performed using Pierce ECL western blotting substrate (Thermo Fisher Scientific) and imaged on a ChemiDoc Gel imaging system (Bio- Rad).

Images were analysed in ImageJ to establish the intensity of staining for each protein and averages calculated for the three independent experiments. T-tests were performed in Excel and statistical significance accepted at p < 0.05.

### Statistical analysis

Tests to determine levels of significance for morphological parameters between different medias for all cell lines were performed using a one way ANOVA in GraphPad Prism. Determination of statistical significance for transcript expression between different media was performed using Tukey’s test as multiple comparisons in GraphPad Prism. Levels of significance were reported as * p<0.05, ** p<0.01, *** p<0.005 and **** p<0.001.

### Ethical approval

Ethical approval for experimental use of hESC lines was given by The University of Queensland Medical Research Ethics Committee.

## Results

### Morphological analysis of hESC cultured in different medias

**Fig 1:**
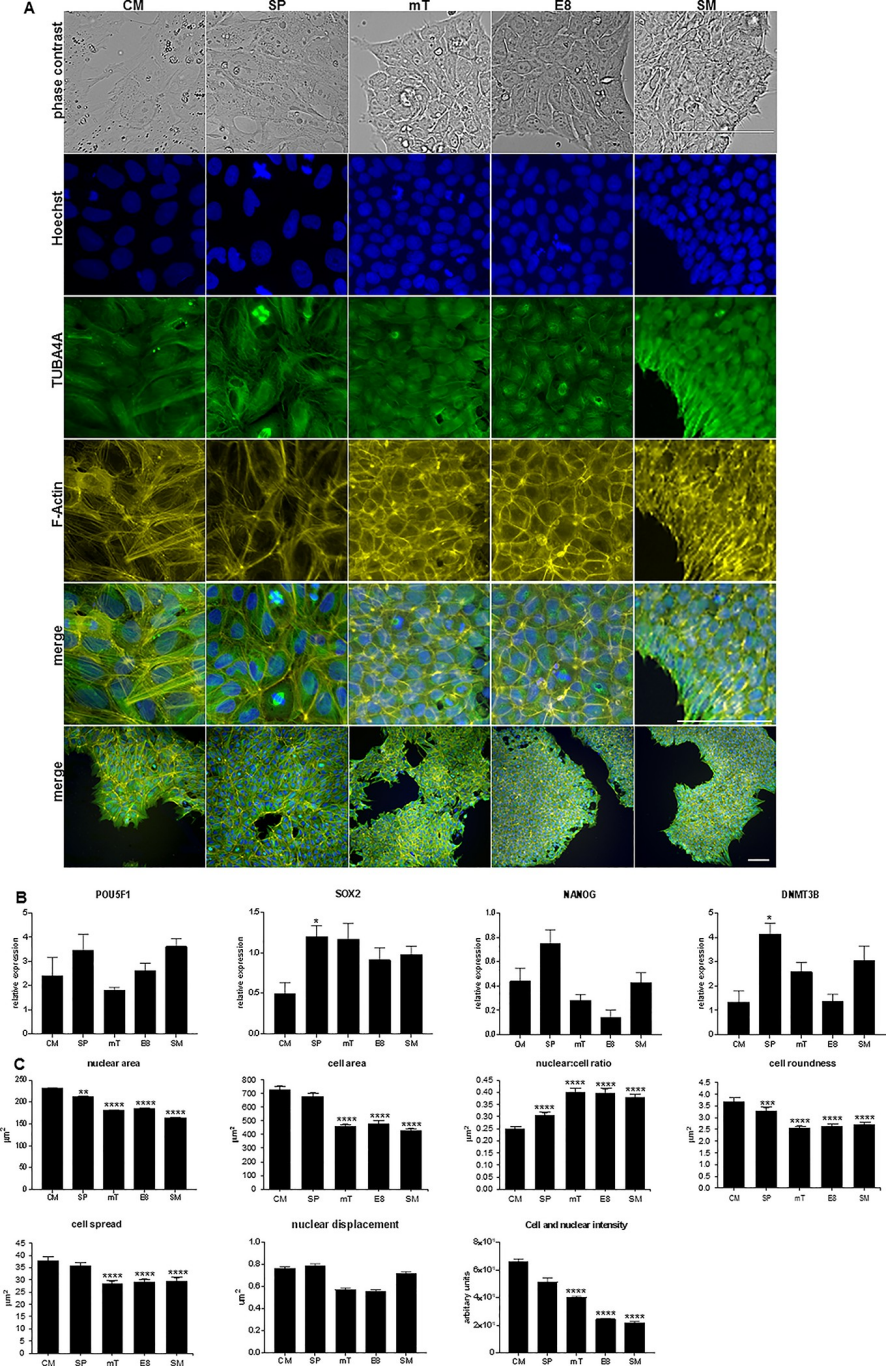
Morphological changes and pluripotency transcript expression in MEL1 cultured in differing medias. (A) Live imaging of MEL1 and staining performed using TUBB4A-488, counterstained with Phalloidin-555 and Hoechst on cells plated in different medias. Differences in colony formation, morphology and F-actin distribution can be observed; lower magnification images are shown for reference; scale bar = 100 μm. (B) RT PCR of pluripotency markers; data presented as mean ± SD with statistical differences shown between CM and other medias; n = 3 independent experiments. (C) Analysis of MEL1 morphological parameters; data presented as mean ± SEM, n = 8 independent experiments. Statistical differences between CM and all other medias are shown in graphs (* p < 0.05; **p < 0.01; ***p < 0.005; **** p < 0.001); statistics for all other parameters can be found in S1 Table.

To investigate observed differences in morphology from hESC cultured in different media, cells were stained with TUBA4A, using Hoechst and Phalloidin as counterstains. Staining for pluripotency markers and gene expression demonstrated that all three cell lines remained undifferentiated (Fig 1B and S1 and S2 Figs).

Cultures performed in CM and SP, were observed to have a looser colony structure and flatter appearance when compared to mT and the Xeno-free medias E8 and SM (Fig 1A, S1A and S2A Figs). Analysis of morphological parameters (Fig 1C, S1B and S2B Figs) demonstrated an overall decrease in nuclear area (NA), cell area (CA), cell roundness (CR) and cell spread (CS) in all three cell lines with the least defined media (CM) demonstrating larger cells, and the Xeno-free medias smaller cells. Levels of significance are reported in S1 Table. HESC demonstrate a high nuclear to cytoplasmic (N:C) ratio [32]. Analysis of our data established that the N:C ratio decreased in MEL1 but increased in both WA09 and ESI-hES3 as media became more defined. This suggests that changes in media composition had a different effect on the remodelling of the cytoskeleton between the cell lines.

Examination of nuclear displacement (ND), the distance between the nucleus’s and cell’s centre of gravity divided by the nuclear spread, demonstrated differences between the three cell lines. WA09 exhibited significant changes in ND between most media with E8 and mT showed increased displacement in comparison to all other media (S1 Fig). ESI-hES3, displayed statistical significance between most media conditions (p < 0.001) with cells cultured in CM demonstrated the greatest, and E8 and SM the least displacement (S2 Fig). No significant difference could be determined in MEL1 between any media. Statistical levels of significance can be found in S1 Table.

Changes in cell and nuclear fluorescence intensity (CNFI) can be detected using the 1xA (N+C) parameter and alterations in F-actin content have previously been correlated with changes in intensity [33]. TGFβis a component of mT [34] and E8 [19] media and it has been reported that when alveolar epithelia cells are supplemented with TGFβ, F-actin content increases [34]. We therefore investigated if analysing CNFI could determine changes between the different medias (Fig 1C, S1B and S2B Figs). MEL1 and WA09 demonstrated significant decreases in intensity as media became more defined. ESI-hES3 also showed a similar trend, however, only SM demonstrated a statistically significant decrease (S1 Table). A previous report has shown that hESC have a more cortical actin profile in comparison with differentiated cells [35]. Here, F-actin distribution, across all cell lines, in CM and SP revealed prominent stress fibres which were not observed in the more defined media (Fig 1B, S1B and S2B Figs). Decreased fluorescence intensity between mT and E8 was only observed in MEL1, with no significant changes observed in WA09 or ESI-hES3, suggesting that the presence of TGFβ in media is not affecting F-actin and therefore stress fibre assembly in either of these cell lines.

Morphological changes in ESC have been observed when cells were cultured on different topographical surfaces and have been shown to influence lineage differentiation [36]. Additionally, it has been reported that morphological, phenotypic and genetic changes in adult mesenchymal (stromal) stem cells (MSC) have been modulated by media composition and were linked to differentiation capacity [37]. Changes in morphology observed here in hESC cultured in different medias suggest that media composition may cause cytoskeletal remodelling. This may result in a more pronounced lineage preference over reported differentiation potential for individual cell lines [2, 9, 10].

### Analysis of morphology in hESC differentiated ST cells cultured in hESC medias

To establish if differences in media composition also affected mesenchymal-like cells differentiated from hESC, we isolated ST cells through withdrawal of FGF, hESC media and MG. We have previously demonstrated that similarly derived cells have mesenchymal properties expressing known human MSC markers and differentiating towards the osteogenic and adipogenic lineages under appropriate conditions [38, 39]. Implantation of these cells has demonstrated the ability to form ectopic bone in vivo [38, 40, 41].

**Fig 2:**
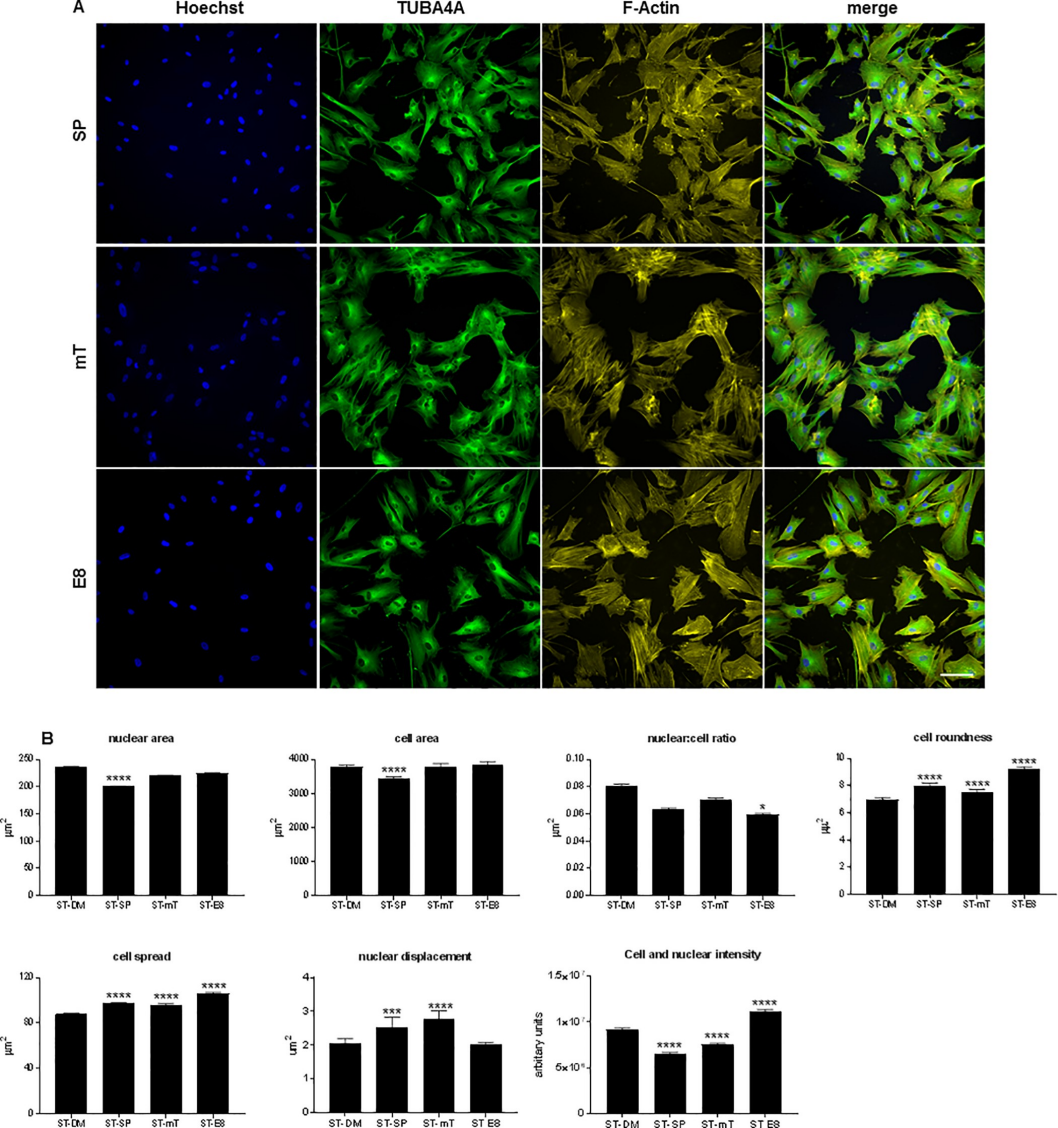
Imaging and analysis of MEL1 ST differentiated cells. (A) MEL1 were differentiated to ST cells in DMEMF/12 with 20% FBS for, minimally, 3 passages and subsequently cultured in SP, mT and E8 media. Staining was performed using TUBBA4A- 488 and counterstained with Phalloidin-555 and Hoechst; scale bar = 100 μm. (B) Analysis of MEL1 ST showing changes in morphological parameters between the different media; data presented as mean ± SEM; n = 3 independent experiments. Statistical differences between CM and all other medias are shown in graphs (* p < 0.05; **p < 0.01; ***p < 0.005; **** p < 0.001); statistics for all other parameters can be found in S2 Table.

ST cells, routinely cultured in DMEM/F12 supplemented with 20% FBS, were transferred to SP, mT and E8 for 2–3 passages. Cells were stained with TUBA4A, Phalloidin and Hoechst to examine if similar changes in altered cell morphology could be determined in differentiated cells. MEL1-ST, WA09-ST and ESI-hES3–ST (Fig 2, S3 Fig) were imaged, morphological parameters (NA, CA, N:C, CS, CR, ND, CNFI) analysed, and a one-way ANOVA performed on the data. Levels of significance can be found in S2 Table.

When ST cells, cultured in DM, SP, mT and E8 media, were compared differences between cell lines response to culture conditions became apparent. Interestingly, WA09 demonstrated an increased number of statistically significant changes in morphological parameters (33/42 changed) between the different media in comparison to MEL1 (27/42) or ESI-hES3 (22/36) (S2 Table). The effect of the media on cell morphology showed increasing size in NA, CA, CR, CS and intensity in CNFI as the media became more defined (SP to E8), as opposed to the decreasing levels observed in the hESC. ST cells cultured in DM media with FBS revealed increased NA, CA, N:C ratio and CNFI as compared to ST cells cultured in SP, mT and E8. These changes in morphology demonstrate the effect of the media on cells irrespective of their cell type. It is, therefore, plausible that reported variations between groups in the transcriptome and thus differentiation potential of hESC [1, 2, 11] may be related, in part, to media composition. Statistically significant changes in CNFI were observed in ST cells cultured in SP, mT and E8 with all 3 cell lines demonstrated decreased intensity in cells cultured in DM and SP. When compared to SP the CNFI increased in both mT and E8 suggesting that the more defined media increased the stress fibre acquisition in differentiated cells.

ST cells, compared with their original hESC line, demonstrated significant changes in all parameters, with increased NA, CA, CS, CR and ND; conversely the N:C ratio decreased (S4 Fig). This is in agreement with previously published data where changes in nuclear structure were investigated in hESC and during early differentiation [42], here the authors demonstrated major differences in the structure of the nucleus as hESC initiated commitment to germ lineages.

These data confirm a previous publication [37] which demonstrated that media composition modulates MSC morphology. We did not investigate if these changes were reflected in differences between cells ability to undergo osteogenic as opposed to adipogenic differentiation as has previously been shown.

### Transcriptional changes in hESC cytoskeleton between different medias

**Fig 3:**
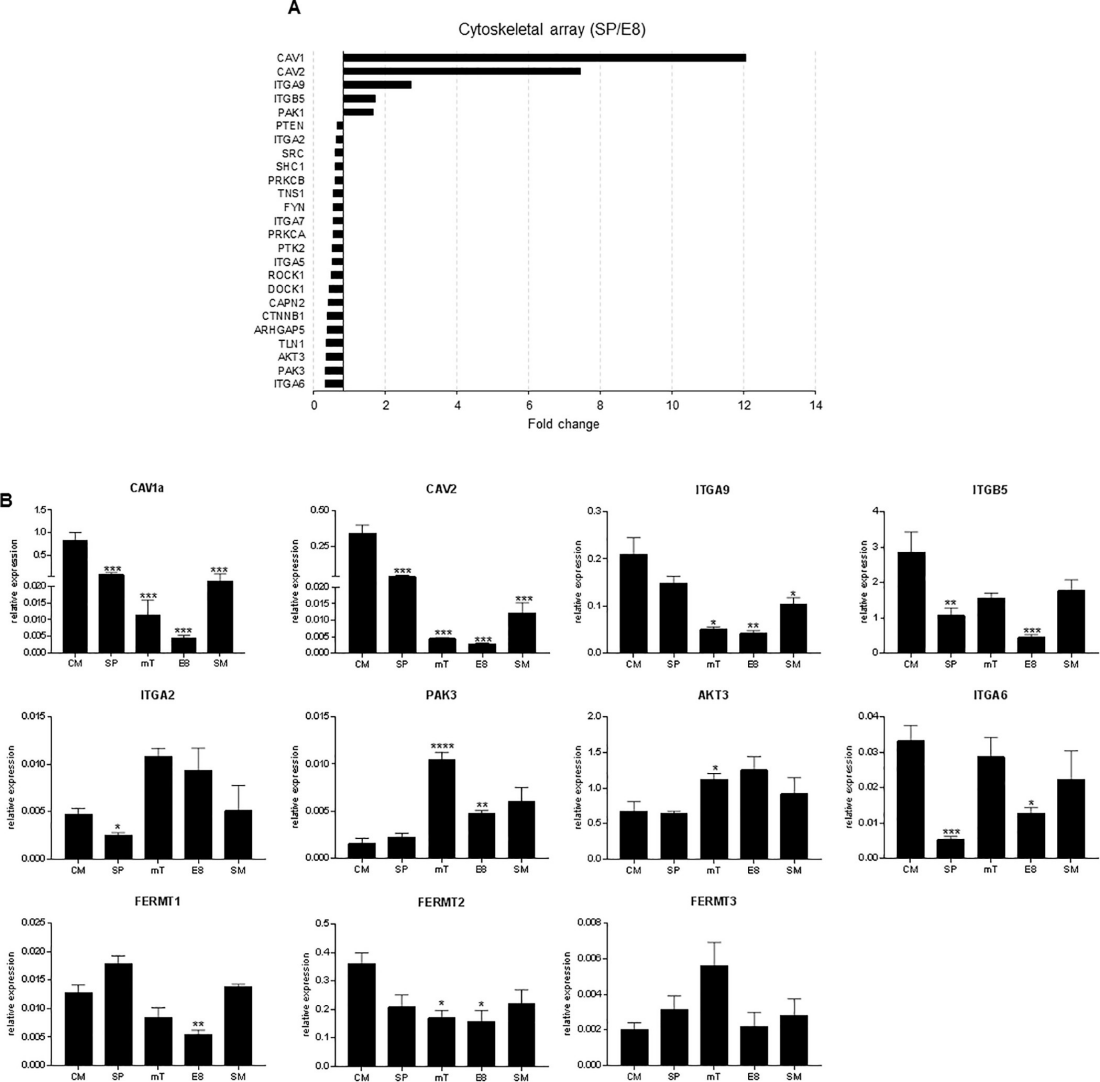
Transcript expression of pluripotency and cytoskeletal for MEL1. (A) Data from RT2 profiler array demonstrating fold change of cytoskeletal genes, up and down regulated over 1.5 fold between SP and E8 (p < 0.05), the majority of genes were up regulated in E8 as compared to SP media for MEL1 (n = 3 independent experiments). (B) RT-PCR validation of selected cytoskeletal genes for 5 hESC medias in MEL1. Data presented as mean ± SD, n = 3 independent experiments. Statistical analysis from multiple t-tests are shown on graphs between CM and all other medias (* p < 0.05; **p < 0.01; ***p < 0.005; **** p < 0.001); statistics for all other parameters can be found in S3 Table.

To investigate changes in cytoskeletal transcript markers in MEL1 cultured in SP and E8, we utilised an RT2 focal adhesion array (Qaigen). Relative expression data were generated using the geometric mean of 5 housekeeping genes, and fold values of SP over E8 calculated with levels of significance accepted at >1.5 fold change, p < 0.05. In the SP medium, CAV1, CAV2, ITGA9, ITGB5 and PAK1 (n = 5) were upregulated. In comparison, an increased number of genes were found to be upregulated in E8 media including ITGA6, AKT3, PAK3, TLN1 and ITGA2 (n = 20; Fig 3A) demonstrating that Xeno media composition significantly affected cytoskeletal remodelling (Fig 3A).

Eight genes identified by the array, either upregulated in SP or E8, were employed to profile all three cell lines cultured in all medias (Fig 3B, S1C and S2C Figs). MEL1 transcript expression of CAV1, CAV2 and ITGA9 all demonstrated decreased expression as the media became more defined (CM to SP to mT to E8). SM (Xeno-free, but composition unknown) appeared to show increased transcript expression, however, the majority of parameters were not found to be significantly different when compared with SP, mT or E8 (S3 Table). These data confirmed, and extended, the RT2 array demonstrating transcript decreases as media become increasingly defined correlating with changes in the morphological parameters NA, CA N:C ratio, CR and CS.

WA09 also demonstrated downregulation of transcript expression for CAV1, CAV2, ITGA9 and ITGB5 between undefined and defined media. Again, this was paralleled in the changes in the NA, CA, CR and CS. CAV1, CAV2, ITGA9 and ITGB5 were observed to have higher transcript levels in SP when compared to CM however, this was not statistically significant. The expression pattern from these genes was mirrored by changes in CNFI suggesting that these genes were associated with changes in the F-actin stress fibre development. In contrast, gene expression of ITGA2, AKT3, PAK3, FERMT1 and FERMT2 demonstrated significant upregulation in SM as compared to the other media.

ESI-hES3 demonstrated a similar transcript profile to MEL1 for CAV1, CAV2, ITGA9 with decreasing transcript levels in association with the more defined media. The transcript profile demonstrated similar decreases with NA, CA, CR and CS between undefined and Xeno-free media. Comparing mT and E8 with SM demonstrated no significant differences in transcript expression except for CAV1 (p < 0.05). Few significant changes were demonstrated in transcript expression of ITGB5, ITGA2, AKT3, PAK3, ITGA6, FERMT1, FERMT2 and FERMT3.

Mechanical cues are acknowledged to be important in the control of cell functions such as differentiation and proliferation. Focal adhesions are responsible for the transfer of mechanical cues from the extracellular matrix to the cytoskeleton [43] and, along with stress fibres, both sense mechanical tension. In addition, stress fibres have routinely been associated with CS [44]. We utilised the same matrix throughout these experiments demonstrating it is therefore plausible that media composition was responsible for the morphological and transcriptional changes observed. The similar decreases between undefined and Xeno-free media detected between some gene expression (CAV1, CAV2, ITGA9) and some morphological parameters (NA, CA, CR, CS) suggests that changes in morphological parameters could be used as a prediction of decreasing expression of these genes.

Multiple T-tests were performed for all parameters, data can be found in S3 Table.

### Changes in transcript expression in different media during early differentiation of MEL1

**Fig 4:**
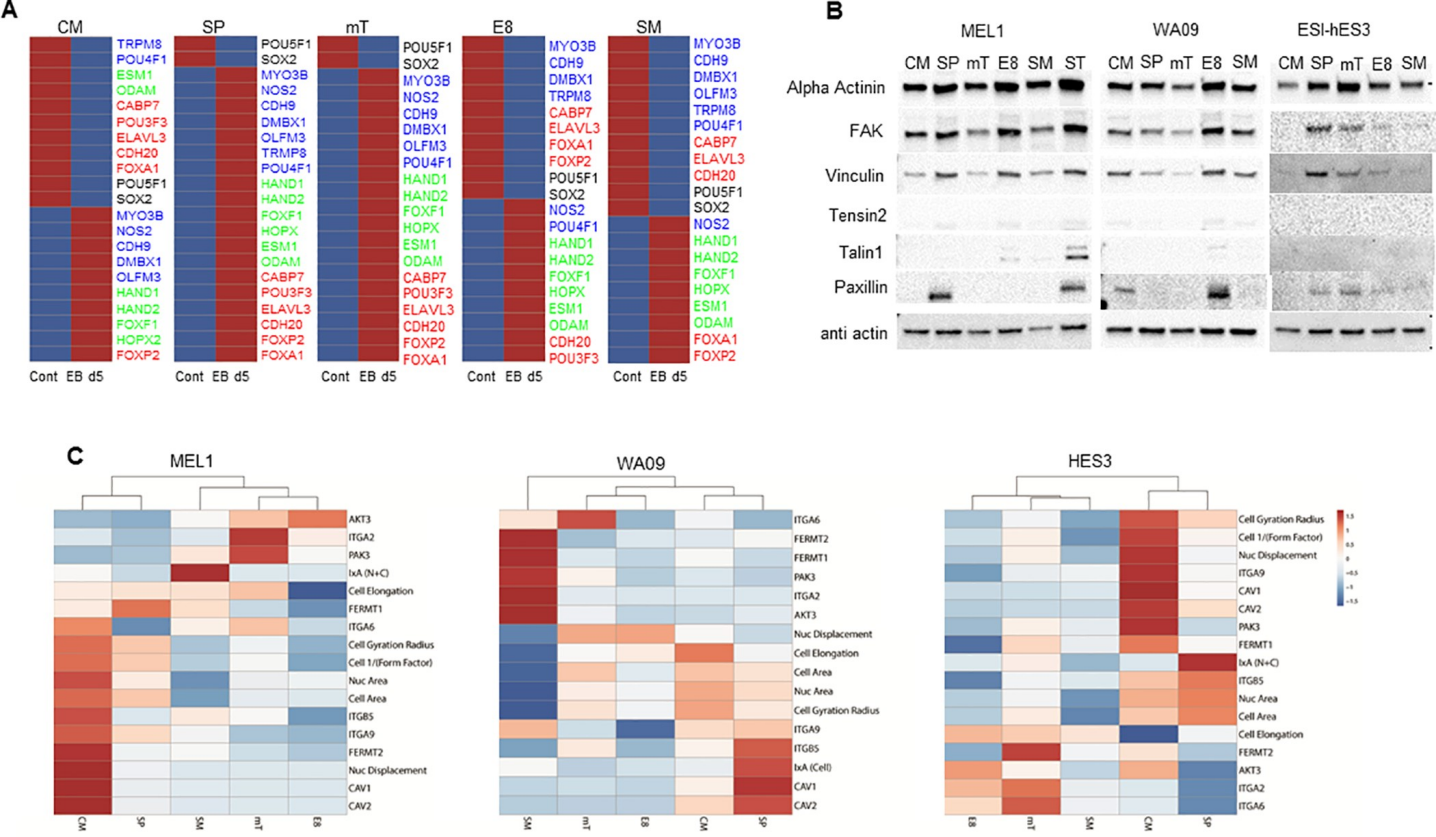
Analysis of protein expression of cytoskeletal markers and differentiation potential of MEL1 cultured in different medias. (A) Differentiation potential of d5 EB in different medias, data suggests SP and mT media have multilineage potential, with CM, E8 and SM showing a more restricted potential. Red bars show upregulated and blue down regulated gene expression as compared to undifferentiated hESC. (B) Western blots showing protein expression of cytoskeletal markers in different medias. (C) Heatmaps of all three hESC lines using morphological parameters and genes utilised for media validation to establish if medias cluster together. For all medias, CM and SP were most similar. Data presented from 3 independent experiments.

MEL1 EB samples for transcript analysis were generated from each media, cultured for 5 days in DMEM/F12, supplemented with 20% FBS. A custom RT2 profiler array was designed for early lineage transcript markers utilising data demonstrated to be upregulated during early differentiation in hPSC [9]. Seven genes for each lineage (endoderm, mesoderm, ectoderm) were selected based on their expression at d5 of EB culture in comparison to hESC samples. The genes included: endoderm (CABP7, POU3F3, PHOXB3, ELAVL3, CDH20, FOXA1, FOXP2); mesoderm (HAND1, HAND2, FOXF1, PLVAP, HOPX, ESM1, ODAM); ectoderm (MYO3B, NOS2, CDH9, DMBX1, OLFM3, TRPM8, POU4F1). Transcript levels of PHOX3B and PVLAP (all samples), POU3F3 (SM), TRPM8 (mT) and OLFM3 (E8) did not demonstrate upregulation in EBs and were omitted from the analysis. ClusterVis was utilised to generate colour maps to compare transcript expression between EBs and control samples for each media (Fig 4A).

All media conditions demonstrated downregulation of pluripotency markers POU5F1 and SOX2 over 1.5 fold between control and d5 EBs. EBs formed from MEL1 cultured in CM showed that mesoderm (4/6; all gene expression > 200 fold) and ectodermal markers (5/7, all gene expression > 1.5 fold) were upregulated in d5 EBs, with only one gene upregulated from the endodermal markers (FOXP2, 64 fold). In comparison, both SP and mT demonstrated upregulation of transcript levels for all three germ lineages (all genes > 1.5 fold). EBs originating from E8 and SM demonstrated upregulation of all mesodermal markers (all upregulated over 6 fold) with only two genes for SM endoderm (FOXA1, POU3F3; > 1.5 fold) and one in ectoderm (NOS2; > 8 fold), and two in E8 (POU4F1, NOS2; > 4 fold) for ectoderm upregulated. No statistical significance was found in transcript levels of undifferentiated hESC between different media.

These data suggest that changes in media composition may impose preferential early lineage differentiation. However, as with previous reports demonstrating variability between cell lines [2, 9, 45], the process by which cells change in different media conditions may be cell line dependant.

### Analysis of cytoskeletal markers by western blot

Integrin α6β1 (ITAG6) has been reported to be the dominant integrin in hESC during differentiation [46]. Vitillo et al reported that inhibition of focal adhesion kinase (FAK) activity in hESC cultured on MG in human cell conditioned media causes the cells to detach and undergo apoptosis or differentiation [47]. Contradictory to this ITGA6 has been reported to inhibit FAK activity and thus maintains pluripotency of hESC [46].

We utilised a focal adhesion staining kit to investigate if western blotting could detect changes in protein expression of cytoskeletal markers in hESC cultured in different media. Western blotting of MEL1, (Fig 4B) demonstrated weak FAK expression in mT and SM when compared with CM, SP and SM. Vinculin showed decreased levels in CM, mT and SM when compared to SP and E8; and Paxillin was only identifiable in SP cultured cells. In comparison, WA09 demonstrated greater expression in E8 for FAK and Paxillin and ESI hES3 only showed weak bands for SP and mT in FAK, Vinculin and Paxillin (Fig 4B). Talin-1 has been reported to be the most important component in integrin based adhesion [48] and has been previously reported in proteomic studies of hESC (MEF and MG cultured) and mouse ESC [49, 50]. Only weak expression of Talin-1 was observed in E8 and SM (MEL1) and E8 (WA09) as compared to the control MEL1 ST cells. Tensin 2 also only demonstrated weak bands in SP, E8 and SM (MEL1), CM, SP, S8 and SM (WA09). No bands were observed in ESI-hES3.

Substantial differences in expression of cytoskeletal proteins were observed between the three cell lines and between their culture in different medias suggesting that remodelling of the cytoskeleton by media composition is changing the interaction and adherence of cells with the ECM and other cells, however, the response is also cell line dependant.

### Cluster analysis of cytoskeletal parameters

To investigate if changes in the cytoskeleton could be grouped based on media type, morphological parameters and transcript data from cytoskeletal markers were uploaded to ClusterVis [51] and heatmaps generated for each cell line. For all cell lines, CM and SP grouped together. MEL1 and WA09 data demonstrated that mT and E8 also clustered, with SM clustering as a secondary order with mT and E8. In comparison, WA09 demonstrated a secondary order of clustering between the CM/SP and mT/E8, with SM only clustering at the next order. ESI-hES3 grouped mT with SM and E8 with mT/SM (Fig 4C).

These data suggest that all cell lines, when cultured in less defined or undefined media (CM and SP), are consistently separate from the more defined/Xeno-free media. Data additionally demonstrates that media composition may alter early differentiation response of MEL1 suggesting that substituting media can specify the lineage and enhance early differentiation capacity.

## Discussion

Changes to cellular morphology have been associated with alterations in cell phenotype, and recent studies have suggested that a change in mechanical properties can show as differentiation or transformation [52, 53]. A number of different cell types (such as breast [54] and prostate [55] cancer) have demonstrated structural changes that specify shape and organisation in the actin cytoskeleton when comparing malignant and non-malignant cells. In adult MSC endpoint differentiation can be modified through remodelling of the cytoskeleton [52], and in hESC two distinct subtypes of pluripotent hESC, within the cell population, have demonstrated different differentiation potential [56].

Using High Content imaging, we have demonstrated that hESC cells, grown in different media formulations, exhibit significantly different cytoskeletal architecture while still maintaining their pluripotent markers. We have also demonstrated that these cells reveal distinct alterations in cytoskeletal transcript expression. Using a validated cytoskeleton profiler array an increased number of genes were found to be upregulated in Xeno-free media and comparison of protein expression of FAK and ITGA6 transcript expression demonstrated that increased gene expression correlated with weaker FAK bands. ITGA6 has been shown to be important in preventing differentiation by inactivation of integrin B1 and FAK signalling [46]. This data suggests that although the less defined and undefined medias maintain pluripotency, differentiation may be easier to initiate, and more generic, in these medias. Our data suggests that it is plausible that media composition can influence early lineage differentiation preferences. As a result of recent landmark publications using single-cell sequencing during early development [57, 58], it been suggested that multi-lineage cell fate propensity is determined by “some selective force or interaction with the environment, rather than just genetic programs” [59].

Stem cell heterogeneity has demonstrable consequences for self-renewal and lineage fate choice with variations found in the consistency of differentiation, viability and morphology. It has been suggested that rather than arising from a mix of cells, heterogeneity occurs from different cell states within the stem cell compartment [60]. Cells cycling in and out of quiescence demonstrate specific timings, and checks within G1 and synchronisation of the cell cycle have shown induction/enhancement of cellular differentiation in mouse ESC [61, 62]. Intrinsic and extrinsic factors, including transcriptional heterogeneity of PSC, has been linked to the propensity for differentiation or self-renewal [63]. Studies have demonstrated that the nutrient microenvironment, such as carbon metabolism, affects cell behaviour and is thus important in maintenance of self-renewal and lineage choice [64]. Even with consistency in experimental setup and data collection maximised, heterogeneity, within and between cell populations, remains an issue with phenotypic heterogeneity of mammalian cells in vitro the rule rather than the exception [65]. While intrinsic factors such a lineage and genetic predispositions define the metabolic networks that cells use this could be modified by altering the nutrient microenvironment [66].

hESC have been morphologically compared to their differentiated progeny [67], parameters have been selected (prominent and abundant nucleoli, less intracellular spacing, fewer differentiating cell nuclei) which characterise undifferentiated cells [68] and algorithms designed to analyse hPSC colony morphology [69]. These non–invasive methods were generated to either identify pluripotent cells during reprogramming or pluripotent colonies during culture. However, these investigations only provide data on the effect media has on colony formation. Changes found in cellular morphology due to media composition or resultant differentiation propensity of hESC is under reported. We have demonstrated that significant morphological changes occur as a result of hESC culture in different medias with the general trend of cells becoming smaller and rounder in Xeno-free, as opposed to, undefined media. This suggests that the undefined media may be creating dynamic instability in the cytoskeleton, with the cytoskeleton becoming more stabilised in Xeno-free media. Quantitative data has demonstrated that actin and microtubule formation are carefully regulated and responsible for cytoskeletal dynamism that characterises proliferating, differentiating and differentiated cells [20, 21].

## Conclusion

In conclusion, we have demonstrated that hESC cultured in defined media show smaller morphology and tighter colony formation. The changes in cell morphology are indicative of preferential lineage choice for undirected EB differentiation of hESC at d5. Xeno-free media displayed increased mesodermal transcript expression when compared to endoderm or ectoderm. SP and mT media demonstrated equivalent lineage differentiation propensity, however undefined CM showed increased differentiation towards mesoderm and ectoderm. Interestingly, very little preference was shown for endoderm specificity and transcript expression levels were low in all conditions. These data suggest that modifying pluripotency media composition can aid in early lineage specificity and pre preparing cells in specific media can be used to support early differentiation events. However, while media composition may play a significant role in remodelling, individual cell lines respond differently.

## Supporting information

S1 Fig Morphological changes and transcript expression of WA09 for pluripotency and cytoskeletal/focal adhesion genes in WA09 cultured in differing medias. (A) Staining was performed using TUBB4A-488, counterstained with Phalloidin-555 and Hoechst. Differences in colony formation, morphology and F-actin distribution can be observed; lower magnification, merged, images are provided to show colony and cell distribution; scale bar = 100 μm. (B) Analysis of morphological parameters demonstrating changes in all parameters; data presented as mean ± SEM, n = 6 independent experiments. One-way ANOVA analysis for these samples can be found in S1 Table. (C) RT-PCR validation of selected cytoskeletal genes and pluripotency markers for WA09 cultured in 5 hESC medias. Data presented as mean ± SD, n = 3 independent experiments. Statistical analysis from multiple t-tests can be found in S1 Table.
(TIF)

S2 Fig Morphological changes and transcript expression of ESI-hES3 for pluripotency and cytoskeletal/focal adhesion genes in ESI-hES3 cultured in differing medias. (A) Staining was performed using TUBB4A-488, counterstained with Phalloidin-555 and Hoechst. Differences in colony formation, morphology and F-actin distribution can be observed; lower magnification, merged, images are provided to show colony and cell distribution; scale bar = 100 μm. (B) Analysis of morphological parameters demonstrating changes in all parameters; data presented as mean ± SEM, n = 6 independent experiments. One-way ANOVA analysis for these samples can be found in S1 Table. (C) RT-PCR validation of selected cytoskeletal genes and pluripotency markers for ESI-hES3 cultured in 5 hESC medias. Data presented as mean ± SD, n = 3 independent experiments. Statistical analysis from multiple t-tests can be found in S1 Table.
(TIF)

S3 Fig Imaging and analysis of WA09 and ESI-hES3 ST cells. (A) WA09 and (B) ESI-hES3 were differentiated to ST cells in DMEMF/12 with 20% FBS for, minimally, 3 passages and subsequently cultured in SP, mT and E8 media. (Ai and Bi) Staining was performed using TUBBA4A-488 and counterstained with Phalloidin-555 and Hoechst; scale bar = 100 μm. (Aii and Bii) Analysis of morphological parameters between the different media; data presented as mean ± SEM; n = 3 independent experiments. One-way ANOVA analysis for these samples can be found in S2 Table.
(TIF)

S4 Fig hESC and ST cell morphological analysis. While nuclear area significantly changed between ST and hESC cell the biggest alterations were in the expansion of the cell area, spread and roundness. Nuclear displacement and the cell nuclear ratio also changed significantly for (A) MEL1, (B) WA09 and (C) ESI-hES3. Data presented as mean ± SEM; n = 3 independent experiments, * p < 0.05; ** p < 0.01; *** p < 0.005; **** p < 0.001.
(TIF)

S1 Table Statistical analysis using one-way ANOVA of hESC morphological parameters. Data showing levels of significance as: n/s = not significant, * p < 0.05, ** p < 0.01, *** p < 0.005, **** p < 0.001; n = 8 (MEL1) or n = 6 (WA09 and ESI-hES3) independent experiments.
(DOCX)

S2 Table Statistical analysis using One-way ANOVA for morphology of hESC stromal derivatives. Levels of significance are: n/s = not significant, * p < 0.05, ** p < 0.01, *** p < 0.005, **** p < 0.001; n = 3 independent experiments.
(DOCX)

S3 Table Statistical analysis of gene expression from RT-PCR using Multiple t tests. n = 3 independent experiments. Levels of significance are: n/s non-significant, * p < 0.05, ** p < 0.01, *** p < 0.005,**** p < 0.001.
(DOCX)
