# Supplementary figures and images for: Media composition modulates human embryonic stem cell morphology and may influence preferential lineage differentiation potential
Source: PLoS One. 2019 Mar 19;14(3):e0213678. doi: 10.1371/journal.pone.0213678 (PMC6424453; doi:10.1371/journal.pone.0213678)

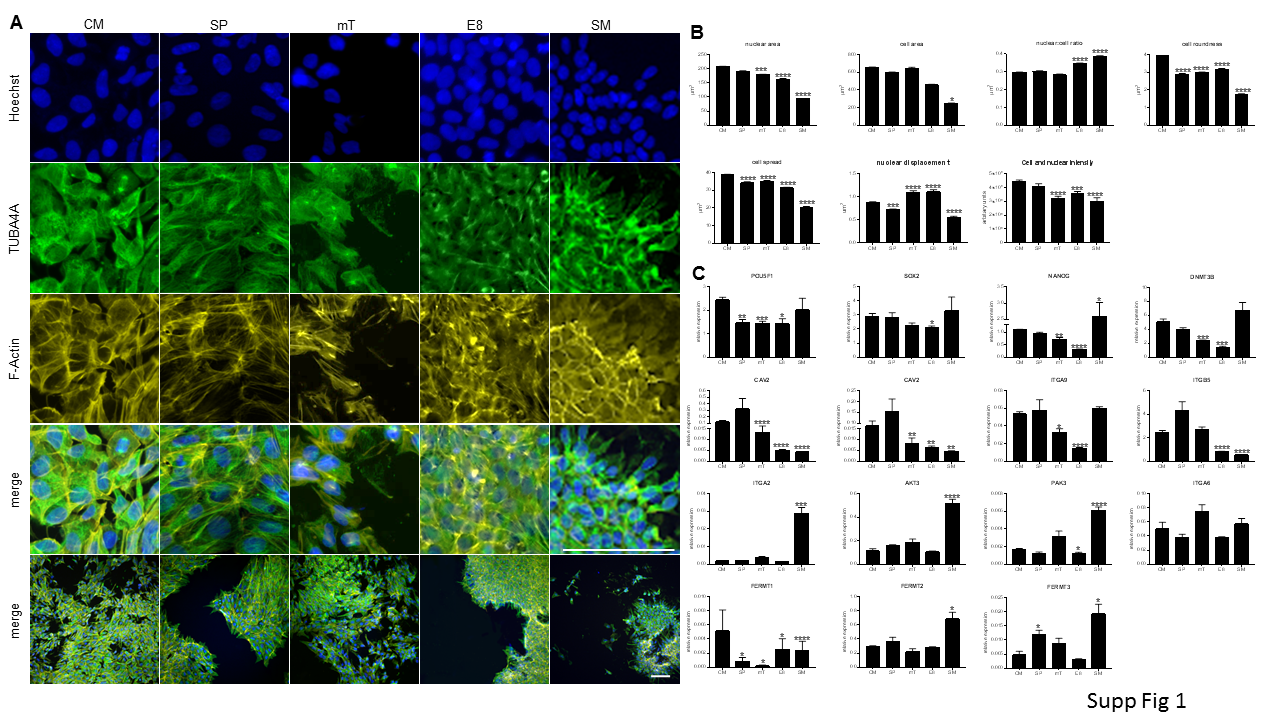

Supplement: S1 Fig — (A) Staining was performed using TUBB4A-488, counterstained with Phalloidin-555 and Hoechst. Differences in colony formation, morphology and F-actin distribution can be observed; lower magnification, merged, images are provided to show colony and cell distribution; scale bar = 100 μm. (B) Analysis of morphological parameters demonstrating changes in all parameters; data presented as mean ± SEM, n = 6 independent experiments. One-way ANOVA analysis for these samples can be found in S1 Table. (C) RT-PCR validation of selected cytoskeletal genes and pluripotency markers for WA09 cultured in 5 hESC medias. Data presented as mean ± SD, n = 3 independent experiments. Statistical analysis from multiple t-tests can be found in S1 Table. (TIF) [file pone.0213678.s001.tif]

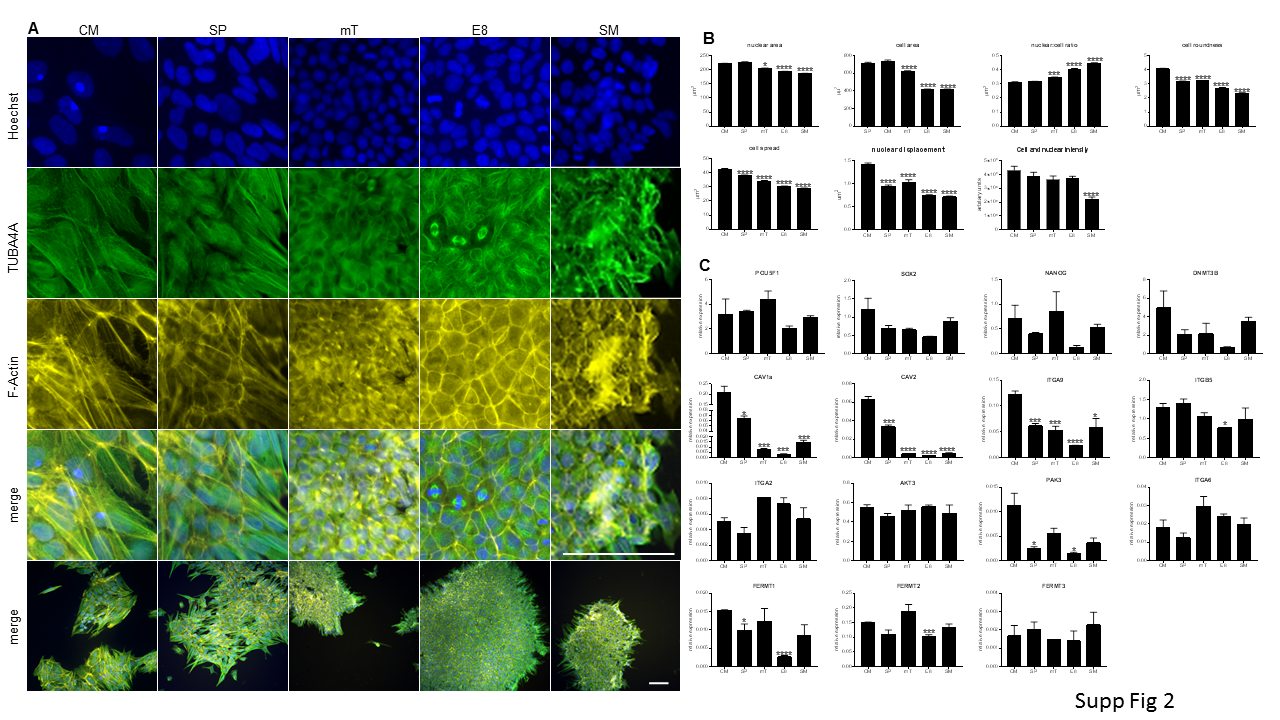

Supplement: S2 Fig — (A) Staining was performed using TUBB4A-488, counterstained with Phalloidin-555 and Hoechst. Differences in colony formation, morphology and F-actin distribution can be observed; lower magnification, merged, images are provided to show colony and cell distribution; scale bar = 100 μm. (B) Analysis of morphological parameters demonstrating changes in all parameters; data presented as mean ± SEM, n = 6 independent experiments. One-way ANOVA analysis for these samples can be found in S1 Table. (C) RT-PCR validation of selected cytoskeletal genes and pluripotency markers for ESI-hES3 cultured in 5 hESC medias. Data presented as mean ± SD, n = 3 independent experiments. Statistical analysis from multiple t-tests can be found in S1 Table. (TIF) [file pone.0213678.s002.tif]

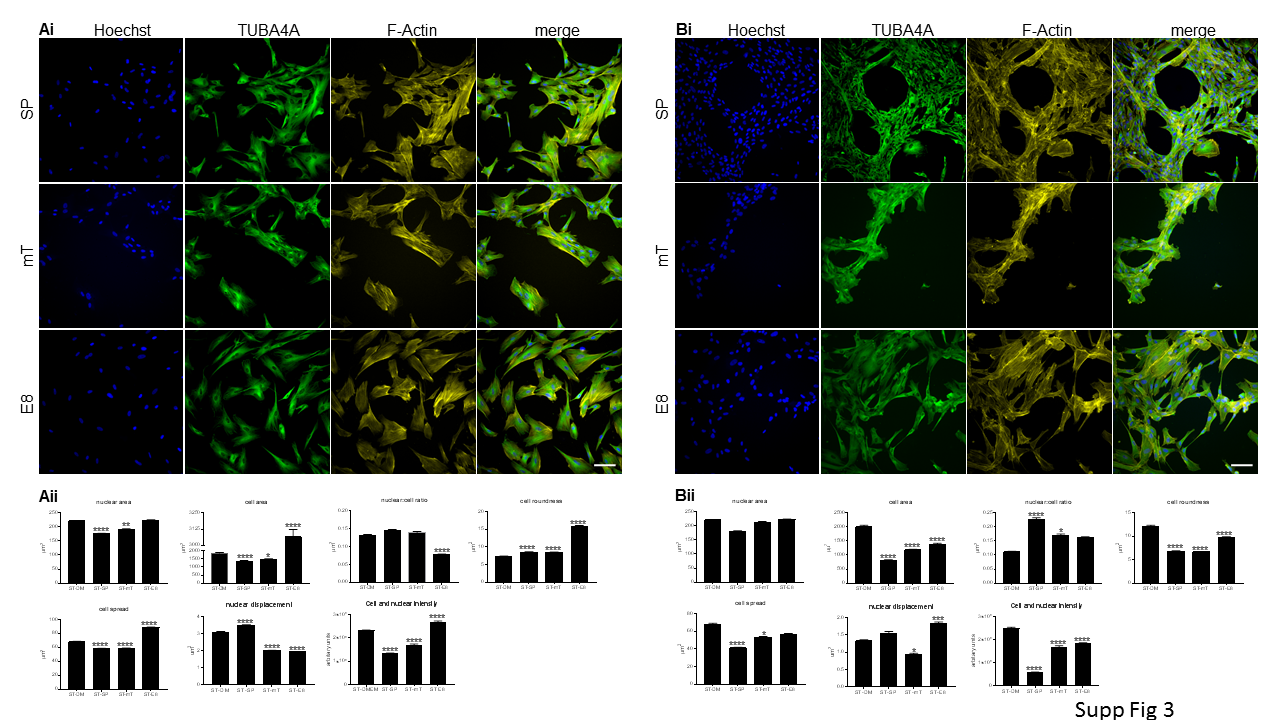

Supplement: S3 Fig — (A) WA09 and (B) ESI-hES3 were differentiated to ST cells in DMEMF/12 with 20% FBS for, minimally, 3 passages and subsequently cultured in SP, mT and E8 media. (Ai and Bi) Staining was performed using TUBBA4A-488 and counterstained with Phalloidin-555 and Hoechst; scale bar = 100 μm. (Aii and Bii) Analysis of morphological parameters between the different media; data presented as mean ± SEM; n = 3 independent experiments. One-way ANOVA analysis for these samples can be found in S2 Table. (TIF) [file pone.0213678.s003.tif]

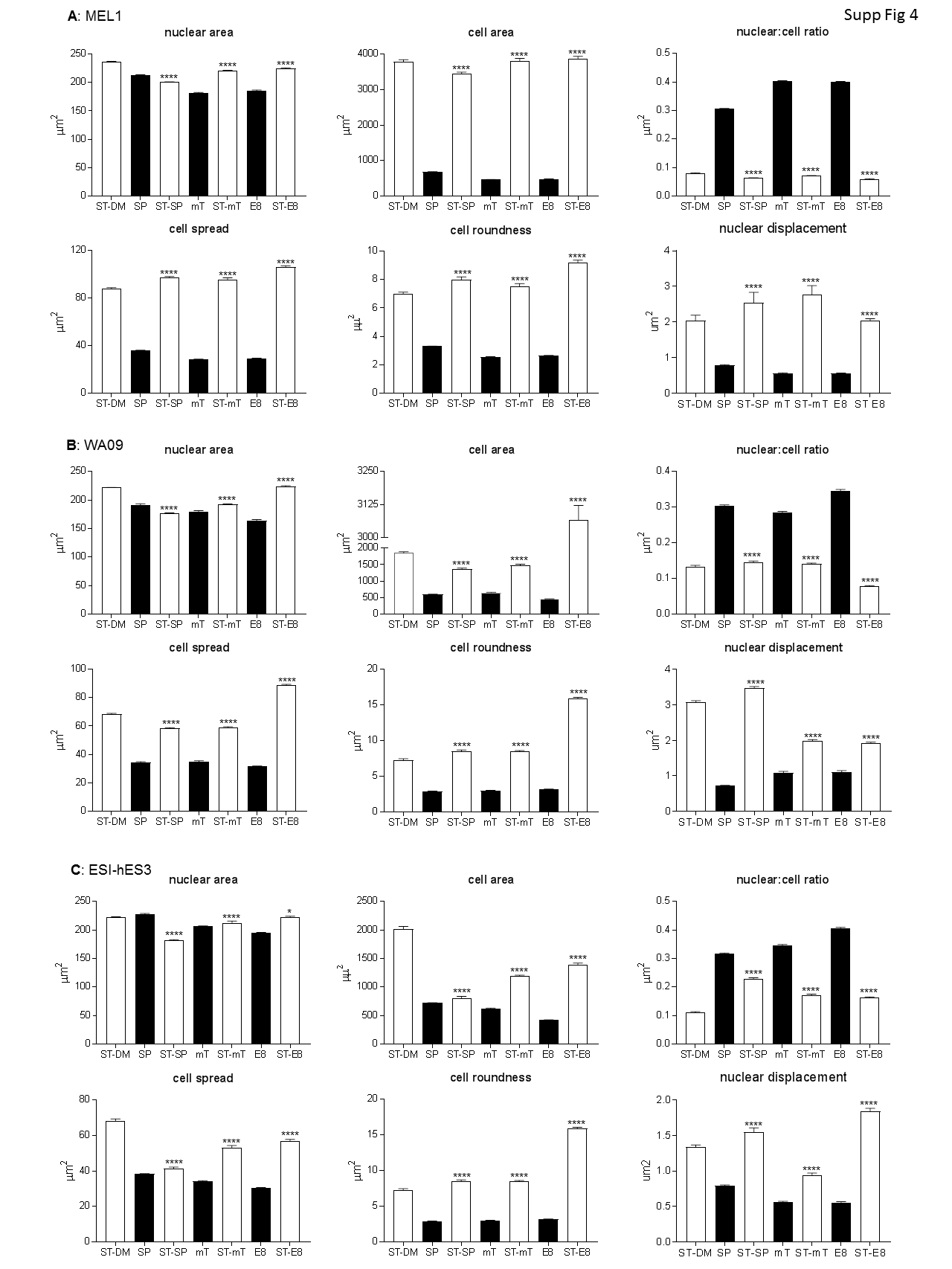

Supplement: S4 Fig — While nuclear area significantly changed between ST and hESC cell the biggest alterations were in the expansion of the cell area, spread and roundness. Nuclear displacement and the cell nuclear ratio also changed significantly for (A) MEL1, (B) WA09 and (C) ESI-hES3. Data presented as mean ± SEM; n = 3 independent experiments, * p < 0.05; ** p < 0.01; *** p < 0.005; **** p < 0.001. (TIF) [file pone.0213678.s004.tif]
