# Supplementary material for: Media composition modulates human embryonic stem cell morphology and may influence preferential lineage differentiation potential
Source: PLoS One. 2019 Mar 19;14(3):e0213678. doi: 10.1371/journal.pone.0213678 (PMC6424453; doi:10.1371/journal.pone.0213678)
Supplement: S1 Table — Data showing levels of significance as: n/s = not significant, * p < 0.05, ** p < 0.01, *** p < 0.005, **** p < 0.001; n = 8 (MEL1) or n = 6 (WA09 and ESI-hES3) independent experiments. (DOCX) [file pone.0213678.s005.docx]

| MEL1 | CM vs SP | CM vs mT | CM vs E8 | CM vs SM | SP vs mT | SP vs E8 | SP vs SM | mT vs E8 | mT vs SM | E8 vs SM |
| --- | --- | --- | --- | --- | --- | --- | --- | --- | --- | --- |
| Nuclear area | ** | **** | **** | **** | **** | **** | **** | n/s | * | ** |
| Cell area | n/s | **** | **** | **** | **** | **** | **** | n/s | n/s | n/s |
| N:C ratio | **** | **** | **** | **** | **** | **** | **** | n/s | * | n/s |
| Cell roundness | *** | **** | **** | **** | **** | **** | **** | n/s | n/s | n/s |
| Cell spread | n/s | **** | **** | **** | **** | **** | **** | n/s | n/s | n/s |
| Nuclear displacement | n/s | n/s | n/s | n/s | n/s | n/s | n/s | n/s | n/s | n/s |
| Cell & nuclear intensity | n/s | **** | **** | **** | **** | **** | **** | **** | **** | n/s |
|  |  |  |  |  |  |  |  |  |  |  |
| WA09 | CM vs SP | CM vs mT | CM vs E8 | CM vs SM | SP vs mT | SP vs E8 | SP vs SM | mT vs E8 | mT vs SM | E8 vs SM |
| Nuclear area | n/s | *** | **** | **** | n/s | * | **** | n/s | **** | **** |
| Cell area | n/s | n/s | n/s | * | n/s | n/s | n/s | n/s | n/s | n/s |
| N:C ratio | n/s | n/s | **** | **** | n/s | **** | **** | **** | **** | **** |
| Cell roundness | *** | **** | **** | **** | n/s | * | **** | n/s | **** | **** |
| Cell spread | **** | **** | **** | **** | n/s | **** | **** | **** | **** | **** |
| Nuclear displacement | *** | **** | **** | **** | **** | **** | n/s | n/s | **** | **** |
| Cell & nuclear intensity | n/s | **** | *** | **** | ** | n/s | **** | n/s | n/s | n/s |
|  |  |  |  |  |  |  |  |  |  |  |
| hES3 | CM vs SP | CM vs mT | CM vs E8 | CM vs SM | SP vs mT | SP vs E8 | SP vs SM | mT vs E8 | mT vs SM | E8 vs SM |
| Nuclear area | n/s | * | **** | **** | **** | **** | **** | * | *** | n/s |
| Cell area | n/s | **** | **** | **** | **** | **** | **** | **** | **** | n/s |
| N:C ratio | n/s | *** | **** | **** | **** | **** | **** | **** | **** | **** |
| Cell roundness | **** | **** | **** | **** | n/s | **** | **** | **** | **** | **** |
| Cell spread | **** | **** | **** | **** | **** | **** | **** | **** | **** | n/s |
| Nuclear displacement | **** | **** | **** | **** | n/s | **** | **** | **** | **** | n/s |
| Cell & nuclear intensity | n/s | n/s | n/s | **** | n/s | n/s | ** | n/s | *** | * |
