# Supplementary material for: Media composition modulates human embryonic stem cell morphology and may influence preferential lineage differentiation potential
Source: PLoS One. 2019 Mar 19;14(3):e0213678. doi: 10.1371/journal.pone.0213678 (PMC6424453; doi:10.1371/journal.pone.0213678)
Supplement: S2 Table — Levels of significance are: n/s = not significant, * p < 0.05, ** p < 0.01, *** p < 0.005, **** p < 0.001; n = 3 independent experiments. (DOCX) [file pone.0213678.s006.docx]

| MEL1 | DM vs SP | DM vs mT | DM vs E8 | SP vs mT | SP vs E8 | mT vs E8 |
| --- | --- | --- | --- | --- | --- | --- |
| Nuclear area | **** | n/s | n/s | ** | **** | n/s |
| Cell area | **** | n/s | n/s | **** | n/s | n/s |
| Nuclear:cell ratio | n/s | n/s | * | n/s | n/s | n/s |
| Cell spread | **** | **** | **** | n/s | **** | **** |
| Cell roundness | **** | **** | **** | **** | **** | **** |
| Nuclear displacement | *** | **** | n/s | n/s | *** | **** |
| Cell & nuclear intensity | **** | **** | **** | **** | **** | **** |
|  |  |  |  |  |  |  |
| WA09 | DM vs SP | DM vs mT | DM vs E8 | SP vs mT | SP vs E8 | mT vs E8 |
| Nuclear area | **** | ** | n/s | n/s | **** | *** |
| Cell area | **** | * | **** | n/s | **** | **** |
| Nuclear:cell ratio | n/s | n/s | **** | n/s | **** | **** |
| Cell spread | **** | **** | **** | n/s | **** | **** |
| Cell roundness | **** | **** | **** | n/s | **** | **** |
| Nuclear displacement | **** | **** | **** | **** | **** | n/s |
| Cell & nuclear intensity | **** | **** | **** | **** | **** | **** |
|  |  |  |  |  |  |  |
| ESI-hES3 | DM vs SP | DM vs mT | DM vs E8 | SP vs mT | SP vs E8 | mT vs E8 |
| Nuclear area | n/s | n/s | n/s | n/s | n/s | n/s |
| Cell area | **** | **** | **** | *** | **** | n/s |
| Nuclear:cell ratio | n/s | n/s | n/s | n/s | n/s | n/s |
| Cell spread | **** | * | n/s | n/s | * | n/s |
| Cell roundness | **** | **** | **** | n/s | **** | **** |
| Nuclear displacement | n/s | * | *** | **** | n/s | **** |
| Cell & nuclear intensity | **** | **** | **** | **** | **** | n/s |
