# Supplementary material for: Media composition modulates human embryonic stem cell morphology and may influence preferential lineage differentiation potential
Source: PLoS One. 2019 Mar 19;14(3):e0213678. doi: 10.1371/journal.pone.0213678 (PMC6424453; doi:10.1371/journal.pone.0213678)
Supplement: S3 Table — Levels of significance are: n/s non-significant, * p < 0.05, ** p < 0.01, *** p < 0.005,**** p < 0.001. (DOCX) [file pone.0213678.s007.docx]

| MEL1 | CM vs SP | CM vs mt | CM vs E8 | CM vs SM | SP vs mT | SP vs E8 | SP vs SM | mT vs E8 | mT vs SM | E8 vs SM |
| --- | --- | --- | --- | --- | --- | --- | --- | --- | --- | --- |
| POU5F1 | n/s | n/s | n/s | n/s | n/s | n/s | n/s | n/s | ** | n/s |
| SOX2 | * | n/s | n/s | n/s | n/s n/s | n/s | n/s | n/s | n/s | n/s |
| NANOG | n/s | n/s | n/s | n/s | * | * | n/s | n/s | n/s | * |
| DNMT3B | * | n/s | n/s | n/s |  | ** | n/s | n/s | n/s | n/s |
| CAV1 | *** | *** | *** | *** | * | * | n/s | n/s | n/s | n/s |
| CAV2 | *** | *** | *** | *** | * | * | * | * | n/s | * |
| ITGA9 | n/s | * | ** | * | *** | *** | n/s | n/s | * | * |
| ITGB5 | ** | n/s | *** | n/s | * | * | * | *** | n/s | ** |
| ITGA2 | * | n/s | n/s | n/s |  | * | n/s | n/s | n/s | n/s |
| AKT3 | n/s | * | n/s | n/s | ** | * | n/s | n/s | n/s | n/s |
| PAK3 | n/s | **** | ** | n/s | **** | *** | n/s | *** | n/s | n/s |
| ITGA6 | *** | n/s | * | n/s | * | * | n/s | * | n/s | n/s |
| FERMT1 | n/s | n/s | ** | n/s | * | *** | n/s | n/s | * | **** |
| FERMT2 | n/s | * | * | n/s | n/s | n/s | n/s | n/s | n/s | n/s |
| FERMT3 | n/s | n/s | n/s | n/s | n/s | n/s | n/s | n/s | n/s | n/s |

| WA09 | CM vs SP | CM vs mt | CM vs E8 | CM vs SM | SP vs mT | SP vs E8 | SP vs SM | mT vs E8 | mT vs SM | E8 vs SM |
| --- | --- | --- | --- | --- | --- | --- | --- | --- | --- | --- |
| POU5F1 | ** | *** | * | n/s | n/s | n/s | n/s | n/s | n/s | n/s |
| SOX2 | n/s | n/s | * | n/s | n/s | n/s | n/s | n/s | n/s | n/s |
| NANOG | n/s | ** | **** | * | n/s | **** | * | ** | ** | *** |
| DNMT3B | n/s | *** | *** | n/s | *** | **** | * | * | *** | **** |
| CAV1 | n/s | **** | **** | **** | * | * | * | n/s | n/s | n/s |
| CAV2 | n/s | ** | ** | ** | n/s | n/s | n/s | n/s | n/s | n/s |
| ITGA9 | n/s | * | **** | n/s | n/s | * | n/s | * | *** | **** |
| ITGB5 | n/s | n/s | **** | **** | n/s | ** | ** | *** | *** | *** |
| ITGA2 | n/s | n/s | n/s | *** | n/s | **** | *** | * | *** | *** |
| AKT3 | n/s | n/s | n/s | **** | n/s | * | **** | * | *** | **** |
| PAK3 | n/s | n/s | * | **** | n/s | n/s | **** | n/s | * | **** |
| ITGA6 | n/s | n/s | n/s | n/s | * | n/s | n/s | * | n/s | n/s |
| FERMT1 | * | * | * | **** | ** | n/s | **** | n/s | **** | **** |
| FERMT2 | n/s | n/s | n/s | * | n/s | n/s | * | n/s | * | * |
| FERMT3 | * | n/s | n/s | * | n/s | ** | n/s | n/s | n/s | * |

| ESI-hES3 | CM vs SP | CM vs mt | CM vs E8 | CM vs SM | SP vs mT | SP vs E8 | SP vs SM | mT vs E8 | mT vs SM | E8 vs SM |
| --- | --- | --- | --- | --- | --- | --- | --- | --- | --- | --- |
| POU5F1 | n/s | n/s | n/s | n/s | n/s | ** | n/s | * | n/s | * |
| SOX2 | n/s | n/s | n/s | n/s | n/s | * | n/s | * | n/s | * |
| NANOG | n/s | n/s | n/s | n/s | n/s | *** | n/s | n/s | n/s | ** |
| DNMT3B | n/s | n/s | n/s | n/s | n/s | n/s | n/s | n/s | n/s | ** |
| CAV1 | * | *** | *** | *** | **** | **** | *** | * | * | * |
| CAV2 | *** | **** | **** | **** | **** | **** | **** | *** | n/s | n/s |
| ITGA9 | *** | *** | **** | * | n/s | *** | n/s | * | n/s | n/s |
| ITGB5 | n/s | n/s | * | n/s | n/s | *** | n/s | * | n/s | n/s |
| ITGA2 | n/s | n/s | n/s | n/s | n/s | * | n/s | n/s | n/s | n/s |
| AKT3 | n/s | n/s | n/s | n/s | n/s | * | n/s | n/s | n/s | n/s |
| PAK3 | * | n/s | * | n/s | n/s | n/s | n/s | * | n/s | n/s |
| ITGA6 | n/s | n/s | n/s | n/s | * | * | n/s | n/s | n/s | n/s |
| FERMT1 | * | n/s | **** | n/s | n/s | n/s | n/s | n/s | n/s | n/s |
| FERMT2 | n/s | n/s | *** | n/s | n/s | n/s | n/s | * | n/s | n/s |
| FERMT3 | n/s | n/s | n/s | n/s | n/s | n/s | n/s | n/s | n/s | n/s |
